# FE65 and FE65L1 share common synaptic functions and genetically interact with the APP family in neuromuscular junction formation

**DOI:** 10.1038/srep25652

**Published:** 2016-05-11

**Authors:** Paul Strecker, Susann Ludewig, Marco Rust, Tabea A. Mundinger, Andreas Görlich, Elisa G. Krächan, Christina Mehrfeld, Joachim Herz, Martin Korte, Suzanne Y. Guénette, Stefan Kins

**Affiliations:** 1 Division of Human Biology and Human Genetics, Erwin-Schrödinger-Straße13, 67663 Kaiserslautern, Germany; 2 Division of Animal Physiology, University of Kaiserslautern, Erwin-Schrödinger-Straße 13, 67663 Kaiserslautern, Germany; 3 Department of Cellular Neurobiology, Technical University of Braunschweig, Spielmannstraße 7, 38106 Braunschweig, Germany; 4 Institute of Physiological Chemistry, University of Marburg, Karl-von-Frisch-Straße 1, 35032 Marburg, Germany; 5 Centre for Molecular Biology (ZMBH) University of Heidelberg, Im Neuenheimer Feld 282, 69120 Heidelberg, Germany; 6 Center for Translational Neurodegeneration Research, UT Southwestern, Dallas, TX, USA; 7 Genetics and Aging Research Unit, MassGeneral Institute for Neurodegenerative Disease, Department of Neurology, Massachusetts General Hospital, Harvard Medical School, Boston, MA, USA

## Abstract

The FE65 adaptor proteins (FE65, FE65L1 and FE65L2) bind proteins that function in diverse cellular pathways and are essential for specific biological processes. Mice lacking both FE65 and FE65L1 exhibit ectopic neuronal positioning in the cortex and muscle weakness. p97FE65-KO mice, expressing a shorter FE65 isoform able to bind amyloid precursor protein family members (APP, APLP1, APLP2), develop defective long-term potentiation (LTP) and aged mice display spatial learning and memory deficits that are absent from young mice. Here, we examined the central and peripheral nervous systems of FE65-KO, FE65L1-KO and FE65/FE65L1-DKO mice. We find spatial learning and memory deficits in FE65-KO and FE65L1-KO mice. Severe motor impairments, anxiety, hippocampal LTP deficits and neuromuscular junction (NMJ) abnormalities, characterized by decreased size and reduced apposition of pre- and postsynaptic sites, are observed in FE65/FE65L1-DKO mice. As their NMJ deficits resemble those of mutant APP/APLP2-DKO mice lacking the FE65/FE65L1 binding site, the NMJs of APLP2/FE65-DKO and APLP2/FE65L1-DKO mice were analyzed. NMJ deficits are aggravated in these mice when compared to single FE65- and FE65L1-KO mice. Together, our data demonstrate a role for FE65 proteins at central and peripheral synapses possibly occurring downstream of cell surface-associated APP/APLPs.

The FE65 protein family members, FE65 and FE65 like proteins (FE65L1, FE65L2) are scaffolding proteins containing two PTB domains and a WW domain that mediate complex formation with a diverse set of proteins^1^. These include Amyloid Precursor Protein (APP) family members, lipoprotein receptors^2^,^3^,^4^, proteins that regulate cellular processes such as cytoskeletal remodeling^5^,^6^,^7^, synaptic vesicular loading and release^8^, calcium homeostasis^8^, signal transduction^9^,^10^, nuclear signaling^11^ and DNA repair^12^.

Characterization of two FE65 knockout (KO) mouse models, generated using different targeting vectors, have provided insights into the biological roles of the FE65 protein^13^,^14^. The isoform specific p97FE65-KO mice results in loss of the p97 isoform and a 5-fold upregulation of an N-terminal truncated soluble isoform (p60), believed to be derived from the same transcript, that is expressed in the cortex at low levels under normal physiological conditions^13^,^14^. Locomotor activity and behavior in the open field test were normal, but deficits in cognitive behavior using non-spatial learning tasks such as temporal dissociative passive avoidance^13^,^15^ and classic fear conditioning^15^ were noted for these mice. In the Morris water maze spatial learning test, only older p97FE65-KO mice (>14 months old) revealed a significant decline in task performance in the training phase and probe trial of the reversal paradigm, whereas young (2–4 months old) mice showed no impairments^13^,^15^. The p97FE65-KO mice also display early-phase long-term potentiation (LTP) deficits elicited *in vivo* by a single 100 Hz train^15^. These data indicate that the p97 isoform of FE65 plays only a minor role in spatial memory formation and may be involved in short-term plasticity. Possible compensation by elevated p60 levels or shared function with related family members may obscure more severe cognitive deficits.

The second FE65 knockout mouse model, lacking both the p97 and p60 isoforms displays deficits in the hanging wire task that are also observed in FE65L1-KO mice^16^. In contrast to the FE65/FE65L1 double knockout (DKO) mice, which exhibit deficits in neuronal positioning and axon outgrowth in the developing cortex, there are no overt morphological abnormalities present in FE65-KO and FE65L1-KO mouse brains^14^.

Thus, a systematic assessment of the contribution of FE65 protein family members to cognitive and motor functions is lacking. Here, we report severe deficits in motor and cognitive behaviors in mice lacking FE65 protein family members. These are associated with neuromuscular junction abnormalities and altered LTP, respectively. Thus, establishing FE65 family proteins as essential synaptic components in mice.

## Results

### Functional redundancy of FE65 and FE65L1 in motor behavior, control of anxiety and activity

For comparative analysis of FE65-KO, FE65L1-KO, and FE65/FE65L1-DKO mice, we used offspring from FE65^+/−^ and FE65L1^+/−^ heterozygous mouse crossings. Behavior analyses were conducted with a cohort of male FE65-KO (n = 12), FE65L1-KO (n = 12) and FE65/FE65L1-DKO (n = 10) mice aged 4–6-month. These behavioral studies initially included 12 FE65/FE65L1-DKO mice, but we found that two mice had developed unilateral eye lens opacity shortly after performing the behavioral tests. We did not detect eye lens opacity in any other mice by visual inspection. Therefore, only these two mice were excluded retrospectively from data analysis for all behavioral studies.

**Figure 1:**
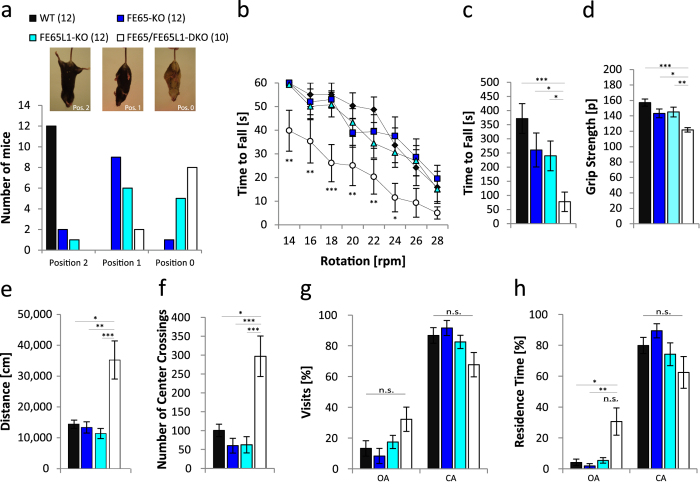
Severe abnormalities in locomotor and anxiety behaviors for FE65/FE65L1-DKO mice. Male FE65-KO (n = 12), FE65L1-KO (n = 12) and FE65/FE65L1-DKO (n = 10) mice (4–6 months-old) were used for all behavioral tests. (**a**) *Paw-clasping response*: Mice were lifted by the tail and the position of their limbs (normal limb position (Pos. 2), front or hind limb clasping (Pos. 1), front and hind limb clasping (Pos. 0) were recorded. (**b**) *Mouse-rotarod*: Mice were placed on a rotating cylinder with different speeds of rotation and the time to fall was measured. Shown is the significance between WT and FE65/FE65L1-DKO mice, since no difference was observed between WT and FE65- or WT and FE65L1-KO mice. (**c**) During the *Hanging Wire Grip Test* the time to fall was measured. (**d**) *Grip strength measurements* of all limbs with a Grip Strength Meter. (**e,f**) *Open-field* behavior recorded for 1 h. (**e**) The distance traveled and (**f)** the number of times mice crossed the center area were measured. (**g,h**) *Elevated Plus Maze* was used to assess anxiety levels. Mice were placed in the closed arm of the plus maze and (**g**) the number of visits in the open arms (OA) and closed arms (CA), as well as (**h**) the residence time was measured over a period of five minutes. Data were analyzed using Kruskal-Wallis-Test followed by Dunn´s Multiple Comparison Test due to differences in normal distribution. Error bars are given as s.e.m. *p < 0.05; **p < 0.01; ***p < 0.001; n.s. for not significant.

Limb clasping in mice briefly held by the tail in an inverted position^17^ showed that the vast majority of FE65/FE65L1-DKO mice (8/10) were unable to adopt the characteristic splayed limb position observed in WT mice (Fig. 1a). The intermediate phenotype, a single set of clasped limbs (position 1)^17^, was observed for the majority of FE65 (9/12) and half of the FE65L1 single KO mice (Fig. 1a). These data are suggestive of motor deficits that may be due to changes in the central nervous system (CNS)^17^, peripheral abnormalities in muscle spindle innervation^18^ or myopathy^19^. No changes in limb position were observed when FE65/FE65L1-DKO mice were held in an inverted position for up to 30 seconds, nor did these mice have difficulty recovering once placed back on a horizontal surface (data not shown). Difficulty with the latter would also be suggestive of CNS deficits^20^. The rotarod test requiring balance and coordination, revealed statistically significant motor deficits only for the FE65/FE65L1-DKO mice (Fig. 1b, p < 0.041). Furthermore in the hanging wire grip test requiring strength and coordination the FE65/FE65L1-DKO mice had a significantly lower latency to fall compared to WT mice, while single KOs, which also showed a trend towards a lower latency to fall, were not significantly different from WT mice (Fig. 1c, p = 0.0001). To directly assess whether motor deficits might involve a peripheral motor defect, the grip strength test was used. Significantly reduced grip strength was observed for the FE65/FE65L1-DKO mice when compared to WT mice, indicating a peripheral motor function deficit in these mice (Fig. 1d, p = 0.00013). Single KO mice showed a trend towards reduced grip strength that was not significantly different from WT (Fig. 1d).

Although FE65/FE65L1-DKO mice showed severe motor deficits, the total traveling distance measured during 1 hour in the open field arena was 2-fold higher than in WT controls, FE65-KO or FE65L1-KO mice (Fig. 1e, DKO vs. WT, p = 0.013; DKO vs. FE65-KO, p = 0.002; DKO vs. FE65L1-KO, p = 0.0001). In contrast to the control and single KO mice, FE65/FE65L1-DKO mice failed to show habituation to this novel environment. Furthermore, FE65/FE65L1-DKO mice crossed the center region more often than the other three genotypes studied (Fig. 1f, DKO vs. WT, p = 0.024; DKO vs. FE65-KO, p = 0.0002; DKO vs. FE65L1-KO, p = 0.0001) (Supplementary Video S1). Given that mice have a natural aversion to open spaces, open field behaviors of the FE65/FE65L1-DKO mice are suggestive of reduced anxiety. To further examine anxiety-like behaviors in these mice, the elevated plus-maze behavioral test was performed^21^. In this test, rodents are exposed to an approach–avoidance conflict between exploratory behavior and their aversion to heights and open spaces. The numbers of visits to the open arm were not significantly different between genotypes (Fig. 1g, p = 0.09). However, FE65/FE65L1-DKO mice displayed a 7-fold increase in the time spent in the open arm of the elevated plus maze compared to WT, FE65- or FE65L1-KO mice (Fig. 1h, DKO vs. WT, p = 0.026; DKO vs. FE65-KO, p = 0.003; DKO vs. FE65L1-KO, p = 0.118). Collectively, these data show that FE65/FE65L1-DKO mice have deficits in peripheral motor function and reduced anxiety. The data also clearly show that FE65 and FE65L1 are functionally redundant in these contexts.

### Learning and memory deficits in FE65/FE65L1-DKO mice

**Figure 2:**
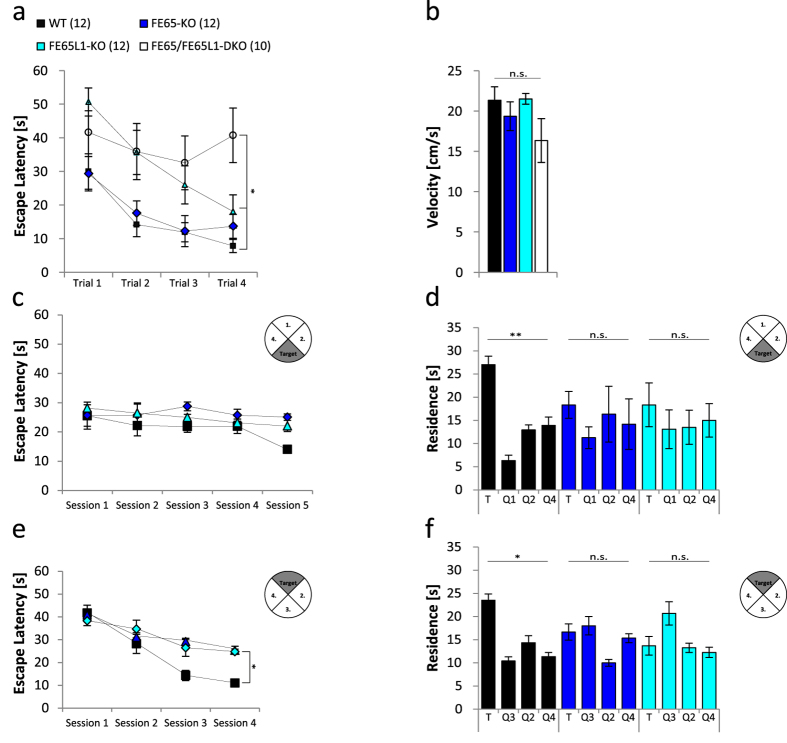
Spatial learning is impaired in FE65-KO and FE65L1-KO mice. The Morris Water Maze (MWM) test was used to examine spatial learning. (**a,b**) FE65-KO, FE65L1-KO and FE65/FE65L1-DKO mice (4–6 months-old) were first trained using a platform placed 0.5 cm beneath the water surface and marked with a black pencil in a pool filled with opaque water. (**a**) Time to find the platform was measured. (**b**) Swim speeds were measured for each mouse by dividing path length by escape time. Shown is the mean velocity of each genotype tested. (**c**) WT, FE65-KO and FE65L1-KO mice were trained for five days in the hidden platform test and the time to find the platform was measured. (**d**) On the 6th day the platform was removed for the probe trial and the time of residence in each quadrant was measured. (**e**) After one day without training, the platform was placed in the opposite quadrant for the reversal paradigm test and the mice were retrained for four days and the time to find the platform was measured. (**f**) On the last day, the platform was removed and the time of residence in each quadrant was measured. The number of male mice (4–6 months-old) is given in brackets. Statistical analysis: (**a,b**) one-way ANOVA with Bonferroni´s post-hoc test. (**d,f**) two-way ANOVA with Bonferroni´s post-hoc test. (**c,e**) two-way repeated measures ANOVA (Greenhouse-Geisser correction). Error bars are given as s.e.m. *p < 0.05; **p < 0.01; n.s. for not significant.

To assess cognitive function, we used the Morris Water Maze (MWM) test. Male FE65-, FE65L1-KO and FE65/FE65L1-DKO mice (4–6-month-old) were initially assessed in the MWM with a visible platform to exclude confounding factors such as deficits in visual perception or swimming performance (Supplementary Video S2). Under these conditions, no differences in procedural learning (Fig. 2a, p = 0.34) or swim speeds (Fig. 2b, p = 0.57) were observed between FE65 and FE65L1 single KOs when compared to WT controls. In contrast, FE65/FE65L1-DKO mice showed normal swim speed but did not learn the task (Fig. 2a, p = 0.042) and appeared disoriented (Supplementary Video S2). We observed strong deficits for the FE65/FE65L1-DKO mice in the MWM tasks. Since interpretation of the cognitive deficits observed for these mice is confounded by altered anxiety, activity and possibly visual abilities^16^ the analyzed data are not further discussed, but are presented in Supplementary Fig. S1.

The FE65- and FE65L1-KO mice were trained for 5 days (4 trials per day) and no significant changes in escape latencies were measured between genotypes (p = 0.56) (Fig. 2c). In the probe trial, WT mice showed a preference for the target quadrant, whereas both FE65- and FE65L1-KO mice lacked a respective preference (Fig. 2d, quadrant 3 (target), WT, p = 0.0085; FE65-KO, p = 0.23; FE65L1-KO, p = 0.46). These data show that genetic deletion of either FE65 or FE65L1 causes spatial memory deficits. After one day without training, the hidden platform was moved to the opposite quadrant of the MWM for the Reverse Hidden Platform test and mice were again trained for 4 days (4 trials per day). During this training phase, the times required to find the platform were now significantly longer for both FE65- and FE65L1-KO mice when compared to WTs (Fig. 2e, p = 0.048). After removal of the platform, FE65- and FE65L1-KO mice lacked a preference for the new target quadrant (Fig. 2f, quadrant 1 (target), WT, p = 0.035, FE65-KO, p = 0.09, FE65L1-KO, p = 0.15). These data indicate that loss of either FE65 or FE65L1 causes deficits in spatial memory retrieval and impaired learning or consolidation in the reversal paradigm.

### Pyramidal neuron spine densities are similar for all FE65 genotypes

**Figure 3:**
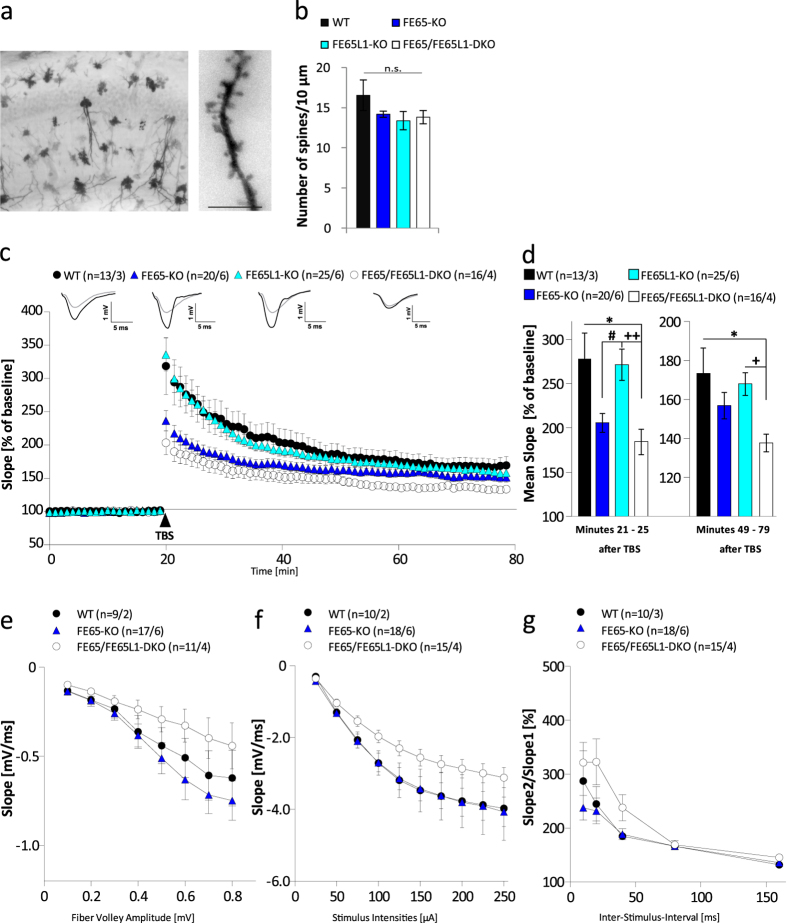
FE65 family KO mice have impaired synaptic plasticity. Coronal slices (100 μm) were used for spine density analysis. (**a**) Overview of Golgi stained neurons in the hippocampal CA1 region of a WT mouse brain. Scale bar represents 10 μm. (**b**) Quantification of total spine density on secondary dendrites of hippocampal CA1 neurons for all genotypes. More than 30 secondary dendrites from three male mice (4–6 months-old) were analyzed for each genotype. (**c–g**) Field excitatory postsynaptic potentials (fEPSPs) from acute hippocampal slices of indicated genotypes were recorded in the CA1 region following stimulation of CA3 Schaffer collateral axons at a frequency of 0.1 Hz. (**c**) LTP was induced by application of theta burst stimulus (TBS) after a 20 min baseline stimulation (arrowhead). The LTP induction rate is shown as percentage (%) of the mean baseline slope. Data points were averaged over 6 time points and error bars indicate s.e.m., n = number of recorded slices/ number of animals. (**d**) PTP values represent averaged potentiation values for the 5 minutes after TBS and the maintenance phase of LTP was calculated from the mean slope of field potentials during the last 30 min of LTP recording. (**e**) Analysis of the Input-Output (IO) strength of FE65 protein family deficient mice. (**f**) Neuronal excitability was tested for all genotypes at increasing stimulus intensities (50–200 μA). (**g**) PPF behavior using 50 ms inter-stimulus-intervals (ISI) was examined for all mouse genotypes. Male mice between four and six month of age were used for spine density analysis and between 8 and 10 month of age for electrophysiology measurements respectively. Data were analyzed using one-way ANOVA with Bonferroni´s post-hoc test. Differences between FE65-KO and FE65L1-KO, between FE65L1-KO and FE65/FE65L1-DKO, and between WT and FE65/FE65L1-DKO mice are marked with #, + , and *, respectively. Error bars are given as s.e.m. *^/ + /^^#^p < 0.05; ^++^p < 0.01; n.s. for not significant.

To investigate whether the observed cognitive deficits are due to altered spine density, we performed Golgi staining of brains isolated from 4–6 month-old WT, FE65-KO, FE65L1-KO, and FE65/FE65L1-DKO mice (see Fig. 3a for representative images). Spine density analyses of second-order CA1 pyramidal neuron dendrites showed a slight reduction compared to WT, but were not statistically significant (Fig. 3b; WT vs. FE65-KO, p = 0.16; WT vs. FE65L1-KO, p = 0.19; WT vs. DKO, p = 0.21). These data suggest that loss of FE65 and/or FE65L1 does not substantially affect neuronal connectivity of hippocampal CA1 neurons.

### LTP, PTP and PPF deficits in hippocampi of FE65 protein family KO mice

The possibility that impaired spatial learning and memory may be due to functional network deficits in the CA3-CA1 synapses was also explored. Extracellular field recordings were performed on acute hippocampal slices obtained from the same cohort of WT, FE65-KO, FE65L1-KO, and FE65/FE65L1-DKO mice used in the behavioral studies. Field excitatory postsynaptic potentials (fEPSPs) were recorded in the CA1 region upon stimulating Schaffer collateral axons in the CA3 at a frequency of 0.1 Hz. LTP was induced via theta burst stimulation (TBS) after 20 min of baseline stimulation and was recorded for 60 min. During these sixty minutes of LTP recording, acute slices of FE65/FE65L1-DKO mice (n = 16/4, corresponding to 16 slices from 4 mice) exhibited lower potentiation compared to WT (p = 0.023, n = 13/3) or FE65L1-KO mice (p = 0.023, n = 25/6) (Fig. 3c). The overall shape of potentiation after TBS application (LTP curve) of FE65L1-KO was similar to that of WT mice (Fig. 3c). The post-tetanic potentiation values (PTP, 5 min after TBS) and the stable phase of LTP were averaged for each genotype. Significantly reduced PTP was observed in FE65/FE65L1-DKO mice when compared to WT mice (184.34 ± 13.97% vs. 277,74 ± 29,15%, p = 0.013) Fig. 3d). FE65-KO mice also showed reduced PTP compared to WT mice, but this was not statistically significant (205.69 ± 10.52% vs. 277,74 ± 29,15%). A significant reduction in the maintenance of LTP, obtained from the mean slope of field potentials during the last 30 min of LTP recording, was observed for FE65/FE65L1-DKO mice in comparison to WT (137.67 ± 4.53% vs. 173.42 ± 12.98%; p = 0.023) (Fig. 3d). Importantly, for FE65-KO mice, only a reduction in PTP was observed, whereas the overall LTP level 60 min after TBS application was only significantly affected in FE65/FE65L1-DKO mice, suggesting partial overlapping functions of FE65 and FE65L1 in synaptic plasticity.

In order to further elucidate whether the LTP defect and lowered PTP observed in FE65/FE65L1-DKO and FE65-KO mice, respectively, were due to altered synaptic transmission, we probed the excitability of hippocampal neurons by increasing the fiber volley (FV) amplitude (Fig. 3e) or the stimulus intensity (Fig. 3f). Analyzing the Input-Output (IO) strength of FE65 protein family deficient mice yielded no alterations between genotypes at any FV amplitude. Although not significant, a trend towards hindered excitability was observed in FE65/FE65L1-DKO FV measurements, with the lowest IO curve of all genotypes (Fig. 3e). Pre-synaptic functionality and short-term plasticity was assessed using the paired-pulse facilitation (PPF) paradigm. Here, none of the analyzed genotypes exhibited any significant difference in PPF values (Fig. 3g). Collectively, these data show that aside from the FE65/FE65L1-DKO mice none of the other FE65 genotypes display significant alterations in basal synaptic transmission.

### FE65 and FE65L1 proteins are required for normal apposition and sizes of pre- and post-synaptic specializations at the NMJ

We observed deficits in motor behavior and more specifically in grip strength for FE65/FE65L1-DKO mice that may result from malformations in the motor cortex, spinal cord or NMJ. Heterotopic neurons were previously found in layer 1 of the motor cortex in the more severely affected adult mice (unpublished data). It is possible that this motor cortex phenotype contributes to the observed motor behavior deficits in a subset of FE65/FE65L1 DKO mice. In contrast, Nissl staining of spinal cord morphology revealed no gross morphological differences between genotypes (Supplementary Fig. S2).

**Figure 4:**
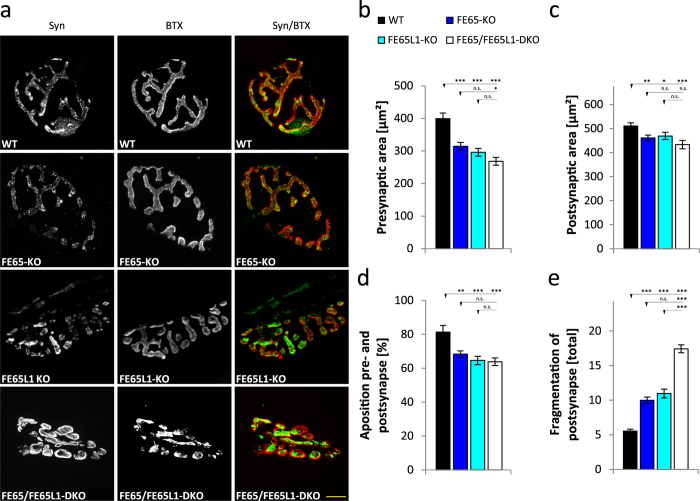
Morphological abnormalities in FE65 family KO Neuromuscular Junctions (NMJs). WT, FE65-KO, FE65L1-KO, FE65/FE65L1-DKO mice were decapitated, the *triangularis sterni* muscle was dissected and the pre- and postsynaptic areas of NMJs were stained using antibodies against Synaptophysin as a presynaptic marker and Bungarotoxin as a postsynaptic marker, respectively. (**a**) Representative pictures of stained presynaptic (Synaptophysin, Syn, green) and postsynaptic areas (Bungarotoxin, BTX, red) in NMJs of *triangularis sterni* muscle for all mouse genotypes studied. Scale bar represents 10 μm. (**b**) Quantification of synaptophysin positive presynaptic areas of *triangularis sterni* NMJs in all FE65 protein family mouse genotypes. (**c**) Quantification of bungarotoxin positive postsynaptic areas of *triangularis sterni* in NMJs of these mice. (**d**) Quantitative analysis of the apposition of synaptophysin and AChR covered areas of *triangularis sterni* NMJs for all FE65 genotypes studied. (**e**) Quantification of postsynaptic fragmentation in *triangularis sterni* NMJs for all FE65 genotypes studied. In total over 30 NMJs from four animals (6–8 months-old) of age of each genotype were analyzed. Statistical analyses were performed using one-way ANOVA followed with Bonferroni´s post-hoc test. Error bars are given as s.e.m. *p < 0.05; **p < 0.01; ***p < 0.001; n.s. for not significant.

NMJ morphology was examined in the *triangularis sterni*. In comparison to other muscle types it offers several advantages: reproducible staining due to its thinness, with fewer than five muscle fiber layers, and the absence of sensory innervation i.e. all neurites branching into this muscle are motor axons^22^. FE65-KO, FE65L1-KO and FE65/FE65L1-DKO mice (6–8 month-old) were examined by staining the pre-synaptic site with an anti-synaptophysin antibody and the post-synaptic site with bungarotoxin, which recognizes nicotinergic acetylcholine receptors (mAChR) (Fig. 4a). Quantification of the pre- and postsynaptic staining showed that the surface area covered by both synaptophysin and AChR were significantly reduced in FE65-KO, FE65L1-KO and FE65/FE65L1-DKO mice compared to WT mice (Fig. 4b, WT vs. FE65-KO, p = 0.0001; WT vs. FE65L1-KO, p = 0.0001; WT vs. DKO, p = 0.0001 and 4c, WT vs. FE65-KO, p = 0.008; WT vs. FE65L1-KO, p = 0.048; WT vs. DKO, p = 0.0008). Pre- and postsynaptic marker apposition was also reduced in the single KO and DKO mice (Fig. 4d, WT vs. FE65-KO, p = 0.002; WT vs. FE65L1-KO, p = 0.0006; WT vs. DKO, p = 0.0002). In addition, the relative number of fragmented NMJs were significantly increased in the FE65- and FE65L1-KOs, and very pronounced in FE65/FE65L1-DKO mice (Fig. 4e, WT vs. FE65-KO, p = 0.0006; WT vs. FE65L1-KO, p = 0.0005; WT vs. DKO, p = 0.00001). Additive effects for FE65 and FE65L1 protein loss were noted for the presynaptic area and the post-synapse fragmentation (Fig. 4b,e). These data suggest that the NMJ morphological defects in FE65 family protein KO mice may be responsible for the muscle strength deficits described above.

### APLP2 deficiency aggravates NMJ deficits in FE65- and FE65L1-KO mice

**Figure 5:**
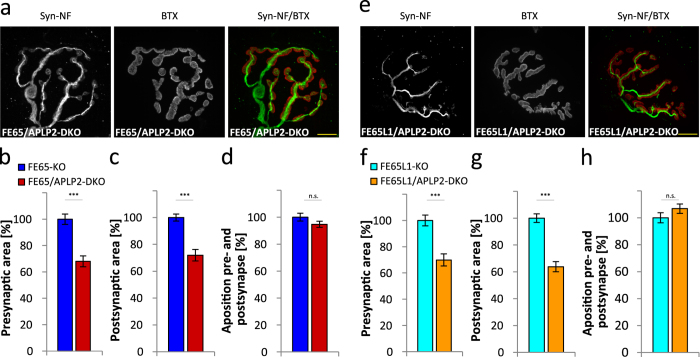
Genetic interaction of FE65 and FE65L1 with APLP2 at the NMJ. FE65-KO, FE65/APLP2-DKO, FE65L1-KO and FE65L1/APLP2-DKO mice were decapitated, the *triangularis sterni* muscle was dissected and the pre- and postsynaptic areas of NMJs were stained using antibodies against synaptophysin as a presynaptic marker, bungarotoxin as a postsynaptic marker and neurofilament H as a marker for axonal tracks, respectively. (**a**) Representative images of stained presynaptic areas, axonal tracks (Synaptophysin and Neurofilament H, Syn-NH, green) and postsynaptic areas (Bungarotoxin, red) of FE65/APLP2-DKO NMJs of *triangularis sterni* muscle. Scale bar represents 10 μm. (**b**) Quantification of synaptophysin-positive presynaptic areas of *triangularis sterni* NMJs in FE65/APLP2-DKO and FE65-KO mice. (**c**) Quantification of bungarotoxin-positive postsynaptic areas of *triangularis sterni* NMJs in FE65/APLP2-DKO and FE65-KO mice. (**d**) Quantitative analysis of the apposition of synaptophysin and AChR covered areas of *triangularis sterni* NMJs in FE65-KO and FE65/APLP2-DKO mice. (**e**) Representative images of stained FE65L1/APLP2-DKO NMJs of *triangularis sterni* muscle. Scale bar represents 10 μm. (**f**) Quantification of synaptophysin-positive presynaptic areas of *triangularis sterni* NMJs in FE65L1/APLP2-DKO and FE65L1-KO mice. (**g**) Quantification of bungarotoxin-positive postsynaptic areas of *triangularis sterni* NMJs in FE65L1/APLP2-DKO and FE65L1-KO mice. (**h**) Quantitative analysis of the apposition of synaptophysin and AChR covered areas of *triangularis sterni* NMJs in FE65L1-KO and FE65L1/APLP2-DKO mice. In total over 30 NMJs from four animals (8 months-old) of each genotype were analyzed. Statistical analyses were performed using the Student´s t-test. Error bars are given as s.e.m. ***p < 0.001; n.s. for not significant.

APP, APLP1 and APLP2 single KO mice showed no changes in NMJ morphology^23^,^24^. Likewise, no change was described for NMJs of APPsα or APPΔCT15 KI mice^25^,^26^. However, when expressed on an APLP2-KO background, the APP, APLP1 null^24^,^27^ as well as APP mutant genotypes lacking the FE65 binding site all produced deficits at the NMJ^24^,^28^,^29^,^30^. To determine whether a similar genetic interaction can be found between APP and FE65 protein family members, we examined the NMJ morphologies in 8-month old FE65/APLP2- (Fig. 5a–d) and FE65L1/APLP2-DKO (Fig. 5e–h) mice. Staining of *triangularis sterni* muscles was performed as described above, with the addition that axonal tracks were visualized with an anti-Neurofilament H antibody (Fig. 5a,e). Quantitation of NMJ specializations revealed significantly smaller surface area staining for the pre- and post-synaptic sites of FE65/APLP2-DKO when compared to FE65-KO mice (Fig. 5b,c; 32%, p = 0.000071 and 28%, p = 0.000018, respectively) and for FE65L1/APLP2-DKO when compared to FE65L1-KO mice (Fig. 5f,g; 30%, p = 0.000016 and 36%, p = 0.000011, respectively). Apposition of pre- and post-synaptic markers (Fig. 5d,h) was also reduced in these mice when compared to WT (p = 0.044), but not when compared to FE65 or FE65L1 single KO mice (p = 0.27and p = 0.25, respectively; data not shown). The existence of a genetic interaction between APLP2 and FE65 or FE65L1 null alleles for NMJ deficits suggests that the physical interaction of these proteins is physiologically relevant at the NMJ.

## Discussion

In this study, we show that mice with genetic deletions of both FE65 and FE65L1 exhibit severe neurological phenotypes, including motor deficits, reduced anxiety, NMJ malformations and LTP impairments. In addition, loss of either FE65 or FE65L1 is sufficient to produce significant spatial learning deficits and NMJ abnormalities. These data suggest widely overlapping functions for FE65 and FE65L1 at central and peripheral synapses. Finally, we demonstrate a genetic interaction between APLP2 and either FE65 or FE65L1 for pre- and post-synaptic NMJ sizes suggesting that the physical interaction of FE65 proteins with APLP2 has functional significance for peripheral synapse structure.

**Figure 6:**
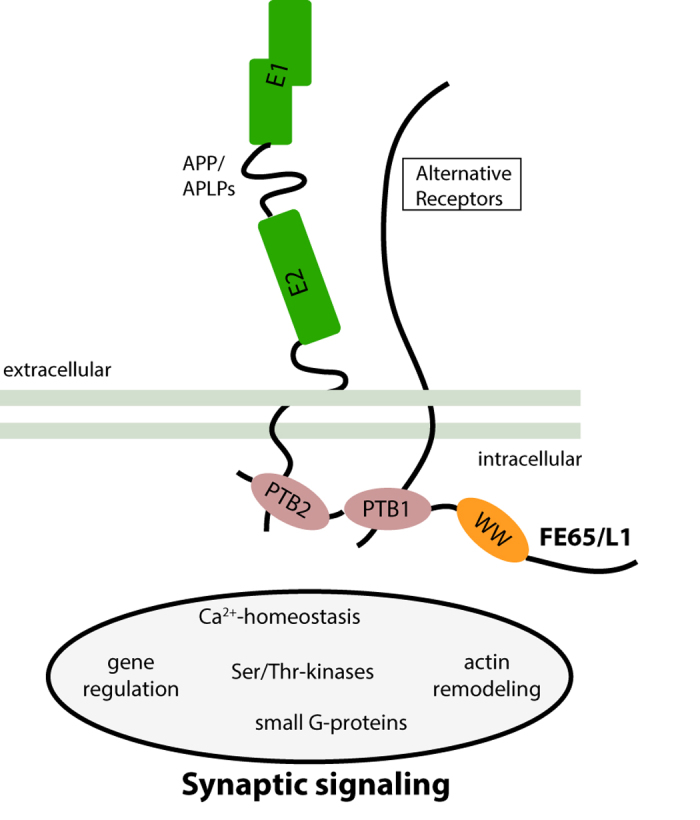
Model summarizing putative FE65/FE65L1 synaptic function. FE65 and FE65L1 are adaptor proteins that bind different receptors (APP, APLP1, APLP2, ApoER2, LRP1, LRP8, VLDLR, Alcadein/calsyntenin, P2x_2_ and others) possibly linking these to pathways that impact synaptic function through molecular interactions with intracellular binding partners, such as calcium signaling (e.g. via DEXRAS1) or actin remodeling (e.g. via Mena) (for a recent review see Chow *et al*.^31^). Based on the remarkable phenotypic similarity of FE65/FE65L1-DKO and APP/APLPs mutants lacking the Fe65 binding site as well as the genetic interaction between APLP2 and FE65/FE65L1 documented in this study, we hypothesize that FE65 and FE65L1 are key components of APP/APLPs synaptic function.

In contrast to the p97FE65-KO, FE65-KO mice have impaired spatial memory^15^. These data suggest that the p60 isoform, which bears both PTB protein-protein interaction domains and a truncated WW domain, is able to compensate for loss of the FE65 protein function involved in spatial memory consolidation or retrieval. Thus, one or more of the 20 proteins with known binding sites in the WW, PTB1 and PTB2 protein-protein interaction domains^31^, such as APP or LRP, may participate in FE65-directed cognitive function (Fig. 6).

FE65L1-KO mice also displayed spatial memory deficits in the MWM. Yet, only FE65/FE65L1-DKO mice showed deficits in the maintenance phase of LTP and only FE65-KO mice showed a trend towards PTP deficits. A subset of aged FE65L1 KO mice (16–20 months old) demonstrated cataract and corneal ulcerations^16^. Thus, we cannot formally exclude the possibility that younger FE65L1 KO mice have some visual acuity deficit that interferes with their performance in the MWM tasks. However, since FE65L1-KO mice behaved similarly to littermate controls in the visible platform task and in the initial training, it appears more likely that loss of FE65L1 affects learning in a manner independent of LTP or visual ability.

A significant difference in the stable phase of LTP (between 50 and 80 min) is only apparent for FE65/FE65L1-DKO mice. Such deficits in the physiological correlate of memory (LTP) are often presented as a cellular mechanism for spatial memory deficits^32^, such as those observed in the MWM for all the KO mice examined in this study. However, since interpretation of the cognitive deficits of FE65/FE65L1-DKO mice is confounded by altered activity (Fig. 1e,f), anxiety (Fig. 1g,h), and possibly visual abilities^16^, LTP deficits in these mice cannot be unequivocally linked to the spatial memory deficits observed in the MWM.

FE65 is reported to play a role in the nucleus that includes regulation of gene transcription^1^,^11^,^33^. Yet, FE65L1 is unable to induce the transcriptional activation of reporter constructs that served to demonstrate this role for FE65^31^,^34^. These data suggest that the molecular mechanism by which FE65 and FE65L1 mediate their effect on LTP is not dependent on gene transcription, since both deletion of FE65 and FE65L1 is required to produce LTP deficits.

A presynaptic role for FE65 and FE65L1 is supported by the observation that expression of both FE65 and FE65L1 is upregulated in mature cortical neurons lacking the three Mint/X11 proteins, which play a critical role in presynaptic neurotransmitter release^35^. However, neither PPF nor basal neurotransmission were significantly affected in FE65-KO or FE65/FE65L1-DKO mice, indicating that neurotransmitter release is unaffected.

Impairments in several motor tasks, rotarod, limb clasping, hanging wire and grip strength tests, were observed in FE65/FE65L1-DKO mice (4–6 months old). FE65- and FE65L1-KO mice also showed a similar trend for deficits that did not reach statistical significance. Notably, significant impairments in the hanging wire test were found in a previous study of 14 months old FE65- and FE65L1-KO mice^16^. The difference between our results and those of Suh and co-workers^16^ may be explained by an additional impact of aging on motor abilities or may be due to different genetic backgrounds. Furthermore, we observed that the size and apposition of NMJ pre- and postsynaptic sites were significantly reduced in FE65- and FE65L1-KO mice and that these deficits were more pronounced in FE65/FE65L1-DKO mice. Thus, the severity of morphological NMJ defects in FE65 family KO mice parallel the muscle strength deficits. Defects at the NMJ may result from loss of function of FE65 and/or FE65L1 at pre- or postsynaptic sites since FE65 and FE65L1 are expressed in neurons and skeletal muscle^14^,^16^,^36^.

As FE65/FE65L1-DKO mice develop brain abnormalities that include cortical heterotopias and hippocampal malformations, which are absent in FE65- and FE65L1-KO mice, it is possible that the brain abnormalities in FE65/FE65L1 DKO mice contribute to the increased latency to fall in the rotarod task^37^, a phenotype unique to FE65/FE65L1 DKO mice.

Histological analyses of the mouse quadriceps muscle from 14 month-old FE65/FE65L1-DKO mice showed an absence of severe muscle degeneration. Notably, 14% of the muscle fiber cells harbor centralized nuclei, usually found in immature myofibers^16^. The decreased apposition of pre- and post-synaptic terminals in FE65 family KO mice may produce partial denervation and subsequent myofiber regeneration that contributes to muscle dysfunction in these mice. Together, our data suggest that the observed motor deficits are predominantly due to NMJ abnormalities, particularly since the severity of NMJ deficits correlate with the extent of the motor deficits.

The phenotypes of FE65/FE65L1-DKO mice resemble those observed for mice expressing APP lacking the FE65/FE65L1 interaction site or carrying a mutation in the FE65/FE65L1 binding site (APP^Y682G^) on an APLP2-KO background^26^,^28^,^38^,^39^, namely impaired locomotor abilities associated with deficits in NMJ formation. Our data showing aggravation of NMJ defects in FE65/APLP2 or FE65L1/APLP2 compound KO mice provide genetic evidence to support the hypothesis that the FE65/APP protein family interactions are essential for NMJ formation. Interestingly, APP interacts biochemically and genetically with LRP4, a key component of the postsynaptic LRP4/MUSK/Agrin complex^40^ mediating signals essential for AChR patterning and stabilization at postsynaptic sites^41^,^42^,^43^. The FE65 protein family members may play a role in modulating LRP4/MuSK/Agrin complex function at the NMJ.

The synaptic function of Mint/X11 proteins^35^ led others to propose a role for these proteins in the manifestation of APP-dependent NMJ phenotypes^28^. However, since the Mint/X11 knockout mice^35^ do not exhibit the same synaptic phenotypes as APP/APLP2 or APLP1/APLP2 knockout mice, and a genetic interaction exists between FE65 or FE65L1 and APLP2, we postulate that of the APP-binding proteins that bind the NPTY motif, it is the FE65 proteins that are primarily responsible for APP/APLP function at the NMJ.

Together our data suggest a pivotal role for FE65/FE65L1 in the central and peripheral nervous systems, possibly downstream of APP/APLP-dependent signaling at the synapse (Fig. 6). These data now form a basis for determining which partners of the FE65/FE65L1 interactome^31^ are involved in these neuronal processes. These may include proteins implicated in nuclear signaling^11^,^44^, actin cytoskeleton regulation^5^,^45^,^46^, or those critically involved in FE65/FE65L1 synaptic function^8^. Alternatively, phosphorylation of FE65 which regulates proteasomal degradation of APP and possibly other binding partners may be responsible for its effects at the synapse^31^.

## Methods

### Animals

FE65- and FE65L1-KO mice^14^ were backcrossed a minimum of ten times on the C57/BL6J (Janvier) mouse background prior to this study. Mice were genotyped as previously described^14^. Ten to twelve male mice (4–6 month-old) of each genotype were used for behavioral studies. WT mice were littermates from crosses set up to generate FE65-KO or FE65L1-KO mice. Mouse husbandry was performed according to local and National Institutes of Health (NIH) guidelines using groups of 4–5 mice per breeding cage, maintained under constant temperature, humidity and a 12 h light/dark cycle, with food and water *ad libitum*. One week before behavioral studies were performed, mice were separated and kept single in breeding cages. Treatment of mice was in accordance with the German law for conducting animal experiments and followed the NIH guide for the care and use of laboratory animals. All experimental protocols were carried out in accordance with the European Communities Council Directive of 24 November 1986 (86/609/EEC). Animal housing, breeding, behavioral studies and euthanasia were approved by the Landesuntersuchungsamt Rheinland-Pfalz,Germany.

### Behavioral studies

All behavioral studies were performed in a blinded fashion.

The Tail Hanging Test^17^ was performed by recording the limb position five seconds after suspending each mouse by the tail. Three different positions were distinguished: Position 2, all limbs were stretched out; Position 1, front or back limbs were tucked; Position 0, all limbs were tucked.

For the Hanging Wire Test, the edges of a home cage wire lid were masked with tape to prevent mice from climbing over the rim onto the top of the wire lid during the experiment. Mice were placed on the middle of the wire lid, which was carefully inverted to ensure that mice had a proper grip on the wires and the latency to fall was measured.

For each mouse, the grip strength was measured for all limbs using a Grip Strength Meter 303500 ®(TSE Systems, v.2.32). Mice were put on a special grip mounted on a force sensor and slowly pulled by the tail. The maximum force exerted was shown on a connected control unit after the animals released their grip.

Movement coordination was analyzed using the Mouse Rota Rod (Ugo Basil, type 7600). Mice were trained for two days at the slowest rotation speed (14 rpm). Two training sessions were performed on each day with a 15 min break in between. On the third day, mice were placed on the Rota Rod and tested using a stepwise increase in rotation speed from 14 to 28 rpm with 2 rpm increases every 60 s and time to fall was measured.

For open field analysis mice were put in a box with an area of 60 cm × 60 cm (TSE Systems) for one hour. Their movement, traveled distance and residence time in the center and in the periphery of the open field were recorded and evaluated with the TSE VideoMot 2 software over the entire 60 min time period^21^.

For the Elevated Plus Maze test^21^ mice were placed on a 50 cm high cross-shaped table (TSE Systems) with two closed and two open arms. Mouse behavior in the maze was recorded for 5 min and the time spent in the open arms was evaluated using the TSE VideoMot2 software.

For Morris Water Maze experiments (TSE Systems)^21^,^47^, mice were first tested for their ability to climb onto the platform and become familiar with the task, mice were trained for one day using a visible platform, identified by a black pencil fixed to the platform in a vertical position. For each trial a single mouse was placed in the water at different locations. On the next day the platform was moved to another quadrant, the pencil was removed and four symbols of different shapes and color were placed on the pool wall for orientation. Mice were trained for five days, 4 trials/day, in a pool filled with opaque water containing a platform placed 0.5 cm beneath the water surface. The probe trial was performed on the sixth day and involved removing the platform and recording the residence time of mice in each quadrant for 60 s. Training for the reverse hidden platform test was started on the eight day after one day of rest. In this test the hidden platform was moved to the opposite quadrant and mice were trained for four days, 4 trials/day. The probe trial for this test involved removing the platform on day 12 and recording the residence time of mice in each quadrant over 60 s. Training and probe trials were recorded and analyzed using the TSE VideoMot 2 Software. The time required for each mouse to find the platform and the residence time in each quadrant was evaluated for the two probe trials.

### Spine density analysis

Male mice (4–6 month old) were deeply anesthetized with 4% chloral hydrate/PBS with 100 μl/10 g bodyweight and perfused with 50 ml PBS followed by 50 ml 4% PFA/PBS to fix the tissues. Brains were dissected and fixed overnight at 4 °C in 4% PFA/PBS. Golgi staining was performed with the FD Rapid Golgi-Staining^TM^ Kit on 100 μm thick coronal slices obtained using a vibratome (Campden Instruments Ltd.). Spines were counted on 10 μm long secondary dendrites of hippocampal neurons of the CA1 basal and apical region.

### Histological analysis

*Triangularis sterni* muscle of male mice (6–8 months-old) were dissected as described^22^. Mice were decapitated after CO_2_ treatment and the muscle was dissected and stored in PBS for subsequent staining. Muscle tissue was incubated with an antibody against synaptophysin (Invitrogen) to stain presynaptic vesicles, anti-neurofilament H (Chemicon) to visualize axons and Alexa594 conjugated bungarotoxin (Invitrogen) to stain nicotinic acetylcholine receptors in the postsynapse.

For spinal cord analysis mice (6–8 months old) were deeply anesthetized with 4% chloral hydrate/PBS using 100 μl/10 g bodyweight and perfused with 50 ml PBS followed by 50 ml 4% PFA/PBS for tissue fixation. Spinal cords were carefully dissected as described^48^ and fixed overnight at 4 °C in 4% PFA/PBS followed by another incubation overnight in 30% Sucrose/PBS at 4 °C. 30 μm thick frozen coronal sections of the ~C8-C6 region were prepared according to Harrison and co-workers^49^ using a freezing microtome (Microm KS 34, Thermo Scientific) and mounted on slides for further analyses. Sections of the spinal cord were Nissl stained for morphological evaluation or sections were immunostained with an antibody against choline acetyltransferase (#178850, Abcam) according to the manufacturer´s protocol to visualize motor neurons in the mouse cervical spinal cord anterior horn^48^.

Images of Nissl stained spinal cord sections were generated with an Olympus SZX7 microscope, an Olympus DP20 camera and the CellF software (Olympus, Hamburg, Germany). Microscopic images for NMJ and spine density analysis were recorded using a Zeiss Axio Observer Z.1 and the corresponding Axio Vision Rel.4.8 software. For quantification of areas, apposition and fragmentation ImageJ software was used.

### Electrophysiology

Acute hippocampal transversal slices were prepared from individual 8 to 10 month-old mice. Mice were anesthetized with isoflurane and decapitated. The brain was removed and quickly transferred into ice-cold carbogenated (95% O_2_, 5% CO_2_) artificial cerebrospinal fluid (ACSF) containing 125 mM NaCl, 2 mM KCl, 1.25 mM NaH_2_PO_4_, 2 mM MgCl_2_, 26 mM NaHCO_3_, 2 mM CaCl_2_, 25 mM glucose. Hippocampi were dissected and cut into 400 μm thick transversal slices with a vibratome (Leica, VT1200S). Slices were maintained in carbonated ACSF at room temperature for at least 1.5 h before transferred into a submerged recording chamber. Slices were placed in a submerged recording chamber and perfused with carbonated ACSF (32 °C) at a rate of 1 to 1.5 ml/min. Field excitatory postsynaptic potentials (fEPSPs) were recorded in stratum radiatum of CA1 region with a borosilicate glass micropipette (resistance 3–15 MΩ) filled with 3 M NaCl at a depth of ∼150–200 μm. Monopolar tungsten electrodes were used for stimulating the Schaffer collaterals at a frequency of 0.1 Hz. Stimulation intensity was adjusted to ∼40% of the maximum fEPSP slope for the 20 minutes baseline recordings. LTP was induced by applying theta-burst stimulation (TBS: 10 trains of 4 pulses at 100 Hz in a 200 ms interval, repeated 3 times). Properties of baseline synaptic transmission were analyzed via input-output (IO) -measurements and short-term plasticity was probed via paired pulse facilitation (PPF). The IO- measurements were performed either by application of a defined current values (25–250 μA) or by adjusting the stimulus intensity to certain fiber volley (FV) amplitudes (0.1–0.8 mV). PPF was performed by applying a pair of two closely spaced stimuli in different inter-stimulus-intervals (ISI) ranging from 10 to 160 ms.

All experiments were performed in a blinded manner. Electrophysiological data were collected, stored and analyzed with LABVIEW software (National Instruments, Austin, TX). The initial slope of fEPSPs elicited by stimulation of the Schaffer collaterals was measured over time, normalized to baseline and plotted as average ± SEM. Analysis of the PPF data was performed by calculating the ratio of the slope of the second fEPSP divided by the slope of the first one and multiplying by 100.

### Statistical analyses

In general one-way ANOVA (Microsoft Office, Excel 2010) was used to assess statistical differences between the four genotypes given that data were normally distributed (Shapiro-Wilk-Test) and variance (Levene-Test) was not significantly (α = 0.05) different. The Bonferroni method was used as a post hoc test (GraphPrism Software, www.graphpad.com). The Kruskal-Wallis-Test followed by Dunn´s Multiple Comparison Test was used to assess statistical differences between the four genotypes given that data weren´t normally distributed or variance was significantly different. MWM probe trials data were analyzed using two-way ANOVA (Microsoft Office, Excel 2010) to compare residence times in different quadrants for all tested genotypes. Escape latencies data during Visible, Hidden and Reversed Platform trials were analyzed by two-way repeated measures ANOVA (Greenhouse-Geisser correction, α = 0.05) using the Real Statistics Resource Packsoftware (Release 3.8, Copyright (2013–2015) Charles Zaiontz. www.real-statistics.com) for comparison of learning over time for all tested genotypes. The unpaired two-tailed Student’s t-test (Microsoft Office, Excel 2010) was used when comparing only two sets of data with normal distributions. All data are shown as standard error of the mean (s.e.m.). Significance was set at *p < 0.05 **p < 0.01 and ***p < 0.001, n.s. for not significant.

## Additional Information

**How to cite this article**: Strecker, P. *et al*. FE65 and FE65L1 share common synaptic functions and genetically interact with the APP family in neuromuscular junction formation. *Sci. Rep*. **6**, 25652; doi: 10.1038/srep25652 (2016).

## Supplementary Material

Supplementary Information

Supplementary Video S1

Supplementary Video S2
