# Supplementary material for: FE65 and FE65L1 share common synaptic functions and genetically interact with the APP family in neuromuscular junction formation
Source: Sci Rep. 2016 May 11;6:25652. doi: 10.1038/srep25652 (PMC4899880; doi:10.1038/srep25652)
Supplement: Supplementary Information [file srep25652-s1.pdf]

Supplementary data for

FE65 and FE65L1 share common synaptic functions and genetically interact with the APP family in neuromuscular junction formation

by

Paul Strecker, Susann Ludewig, Marco Rust, Tabea A. Mundinger, Andreas Görlich, Elisa G. Krächan, Christina Mehrfeld, Joachim Herz, Martin Korte, Suzanne Y. Guénette and Stefan Kins

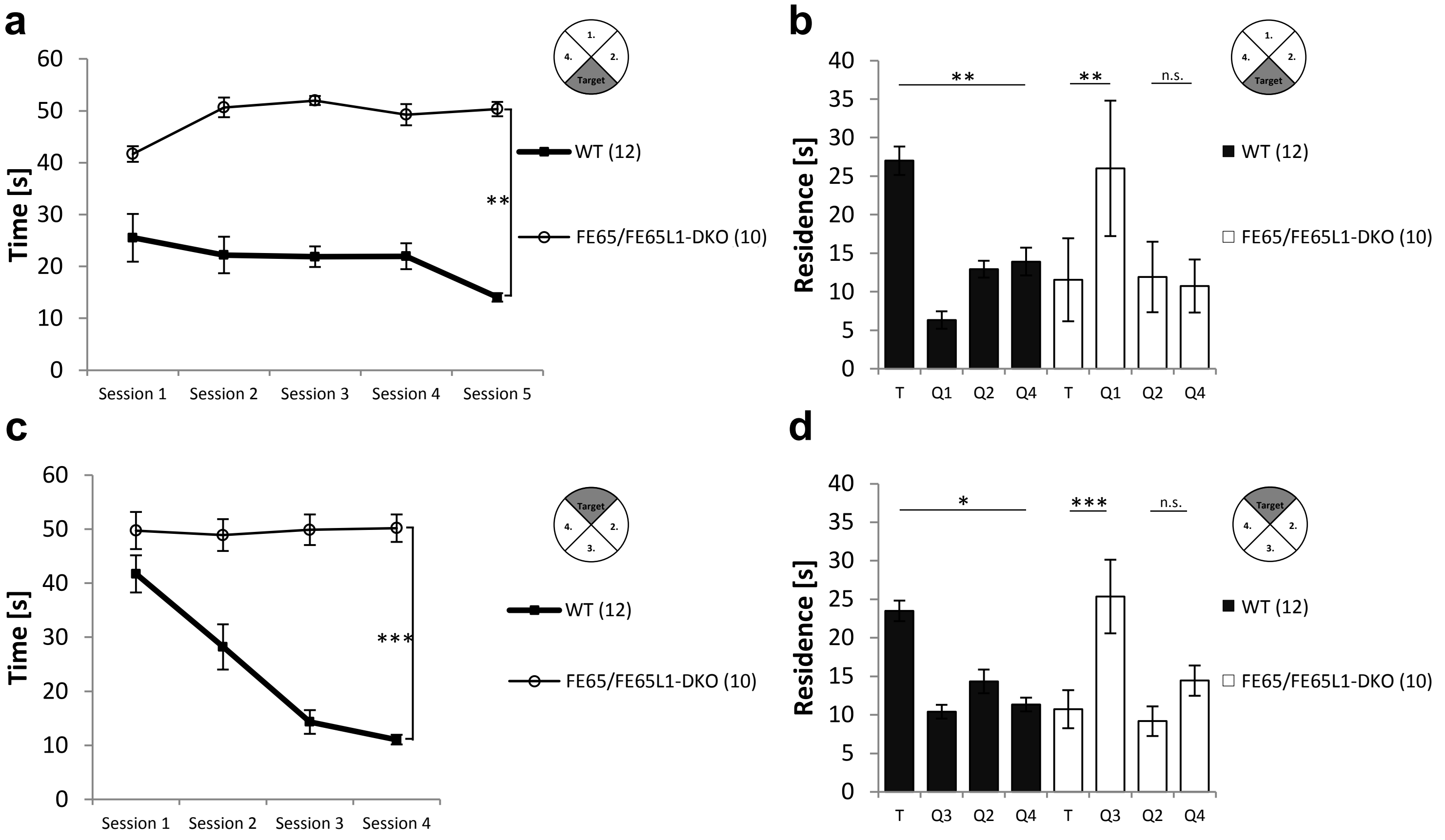

Supplementary Figure S1: Morris Water Maze analysis of FE65/FE65L1-DKO mice. (a) Mice were trained for five days in a pool filled with opaque water. The platform was located 0.5 cm beneath the water surface and the time to find the platform was measured. **(b)** On the 6th day the hidden-platform probe trial was performed by removing the platform and measuring the time of residence in each quadrant. **(c)** After one day without training, the platform was placed in the opposite quadrant and the mice were trained in this new setting for four days. **(d)** On the last day the platform was removed for the reversal learning probe trial and the time of residence in each quadrant was measured. The number of mice is given in brackets. Data from learning phases (Hidden Platform and Reverse Hidden Platform) were analyzed by two-way repeated measures ANOVA (Greenhouse-Geisser correction). Probe trial data were analyzed by two-way ANOVA followed by Bonferroni´s post-hoc test. Error bars are given as s.e.m.. * p< 0,05; ** p< 0,01; *** p< 0,001; n.s. for not significant.

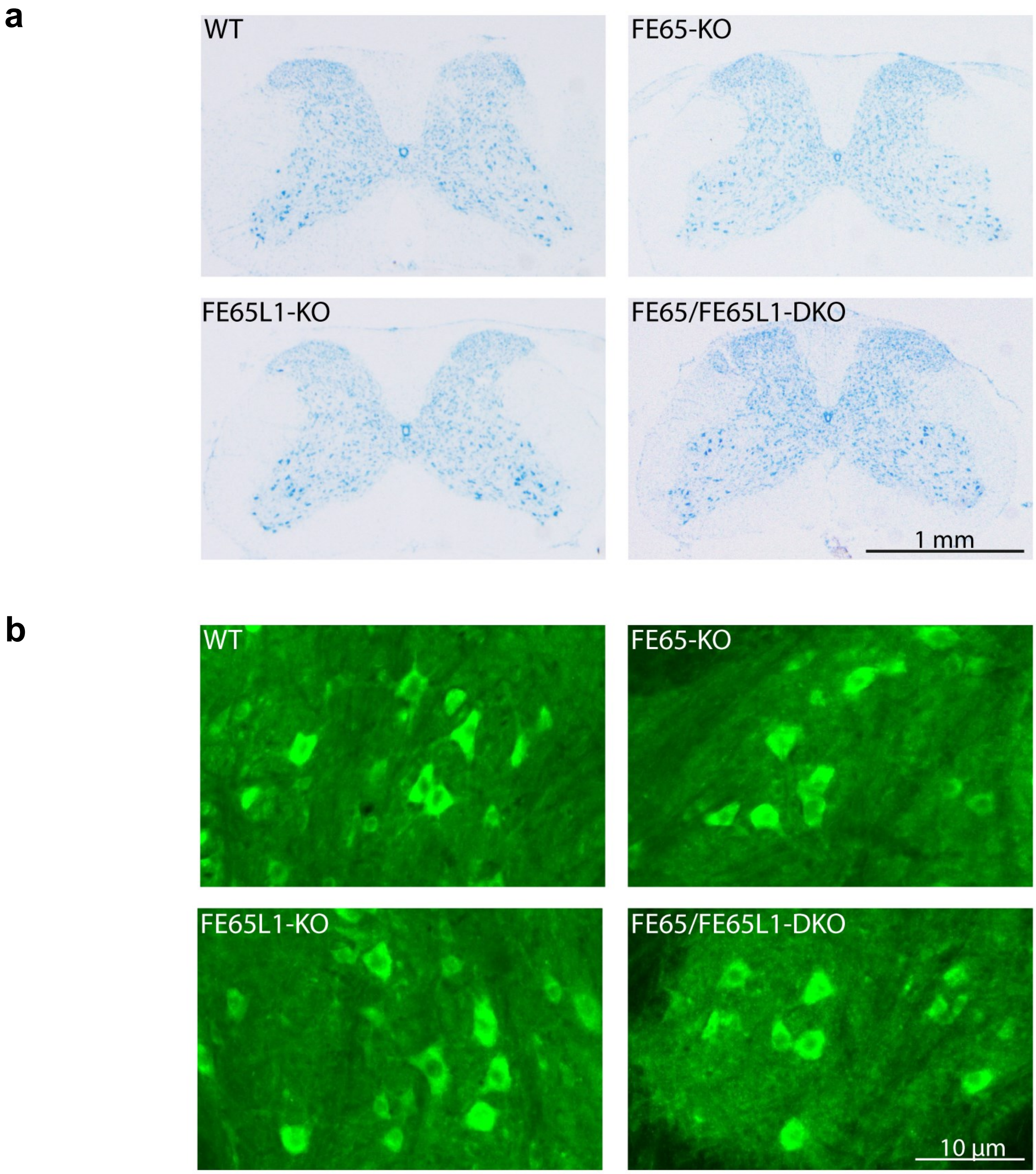

Supplementary Figure S2: No obvious morphological and neuronal changes in spinal cord stainings of FE65 family KO mice. (a) Nissl staining of coronal sections of spinal cord segment ~C8-C6 of 6-8 month old WT, FE65-KO, FE65L1-KO and FE65/FE65L1-DKO mice. **(b)** Choline acetyltransferase staining (#178850, Abcam) of motor neurons in the anterior horn of coronal sections of spinal cord segment ~C8-C6 of 6-8 month old WT, FE65-KO, FE65L1-KO and FE65/FE65L1-DKO mice.

Supplementary Video S1: Open-field behavior of WT and FE65/FE65L1-DKO mice. The behavior of WT, FE65-KO, FE65L1-KO and FE65/FE65L1-DKO mice was recorded for 1h. In this video several sequences (after 30 seconds as well as 10, 30 and 50 minutes) of the recording (1h) from a representative WT and FE65/FE65L1-DKO mouse in the open field is shown.

Supplementary Video S2: Behavior of WT and FE65/FE65L1-DKO mice in the visible platform version of the Morris water maze test. FE65-KO, FE65L1-KO and FE65/FE65L1-DKO mice (4-6 months-old) were trained using a platform placed 0.5 cm beneath the water surface and marked with a black pencil in a pool filled with opaque water. This video shows behavior of a representative WT and FE65/FE65L1-DKO mouse in the visible platform version of the MWM test during the 4th trial. Notable, FE65/FE65L1-DKO mice appeared disoriented.
